# How to Clean and Safely Remove HF from Acid Digestion Solutions for Ultra-Trace Analysis: A Microwave-Assisted Vessel-Inside-Vessel Protocol

**DOI:** 10.3390/mps5020030

**Published:** 2022-04-03

**Authors:** Marco Pinna, Arianna Signorelli, Gilberto Binda, Carlo Dossi, Laura Rampazzi, Davide Spanu, Sandro Recchia

**Affiliations:** 1 Department of Science and High Technology, University of Insubria, Via Valleggio 11, 22100 Como, Italy; mpinna@uninsubria.it (M.P.); asignorelli@studenti.uninsubria.it (A.S.); 2 Norwegian Institute for Water Research (NIVA), Gaustadalléen 21, 0349 Oslo, Norway; gilberto.binda@niva.no; 3 Department of Theoretical and Applied Sciences, University of Insubria, Via Dunant 3, 22100 Varese, Italy; carlo.dossi@uninsubria.it; 4 Department of Human Sciences and Innovation for the Territory, University of Insubria, Via Sant’Abbondio 12, 22100 Como, Italy; laura.rampazzi@uninsubria.it

**Keywords:** hydrofluoric acid, microwave-assisted acid digestion, soil, silicate dissolution, evaporation, ultra-trace elements, ICP-MS, vessel-inside-vessel, green analytical chemistry

## Abstract

The complete dissolution of silicate-containing materials, often necessary for elemental determination, is generally performed by microwave-assisted digestion involving the forced use of hydrofluoric acid (HF). Although highly efficient in dissolving silicates, this acid exhibits many detrimental effects (e.g., formation of precipitates, corrosiveness to glassware) that make its removal after digestion essential. The displacement of HF is normally achieved by evaporation in open-vessel systems: atmospheric contamination or loss of analytes can occur when fuming-off HF owing to the non-ultraclean conditions necessarily adopted for safety reasons. This aspect strongly hinders determination at the ultra-trace level. To overcome this issue, we propose a clean and safe microwave-assisted procedure to induce the evaporative migration of HF inside a sealed “vessel-inside-vessel” system: up to 99.9% of HF can be removed by performing two additional microwave cycles after sample dissolution. HF migrates from the digestion solution to a scavenger (ultrapure H_2_O) via a simple physical mechanism, and then, it can be safely dismissed/recycled. The procedure was validated by a soil reference material (NIST 2710), and no external or cross-contamination was observed for the 27 trace elements studied. The results demonstrate the suitability of this protocol for ultra-trace analysis when the utilization of HF is mandatory.

## 1. Introduction

Microwave-assisted sample digestion is an important routine technique for the effective and safe dissolution of solid samples and subsequent elemental composition or isotope ratio analyses of a wide variety of matrices such as soils [1,2,3], sediments [4,5], minerals [1,6,7] and biota [8,9,10].

Generally, the solid samples are digested using a mixture of pure acids inside a sealed polymeric container (commonly made of fluoropolymers such as Perfluoroalkoxy Alkanes (PFA) and Polytetrafluoroethylene (PTFE)) under microwave (MW) irradiation, to reach elevated temperature and pressure which favor matrix dissolution. The choice of the acid mixture is key for fast and effective digestion and strictly depends on the nature of the materials to be dissolved and the analytes to be determined. For instance, silicate-containing samples normally require the presence of hydrofluoric acid (HF) for quantitative MW digestion, as it is able to efficiently dissolve silicates [2,4,6,11]. Moreover, HF is a strong complexing agent which can also increase the solubility and stability of several metals. Nonetheless, some drawbacks are related to the use of this acid when aiming at the determination of trace elements. The use of hydrofluoric acid may, in fact, lead to the considerable underestimation of a variety of other metals (e.g., Al, Ba, Ca, Mg and Se) due to the formation of relatively insoluble fluoride-based compounds [4,12,13]. This phenomenon may also lead to the coprecipitation of some rare earth elements [14]. Moreover, the employment of HF poses serious instrumental problems when sensitive analytical techniques such as Inductively Coupled Plasma-Mass Spectrometry (ICP-MS) or Inductively Coupled Plasma-Optical Emission Spectrometry (ICP-OES) are adopted: HF is corrosive for both the conventional nebulizing system and the plasma torch since these components are commonly made of glass or quartz. One way to overcome this problem implies the replacement of the entire sample introduction glassware with specific plastic-based equipment (e.g., PFA nebulizer, spray chamber and connector and Pt injector for ICP systems) to avoid any damage due to the residual presence of HF in the solution to be analyzed after digestion: the drawback of this approach is a significant decrease in instrumental sensitivity, probably due to the poorer quality of the generated aerosol [15].

The other strategy to solve this problem implies the removal of HF by evaporation. This method is normally preferred, as the addition of other concentrated acids (necessary to displace HF) allows to redissolve insoluble fluorides improving the overall elements recovery [6,16,17]. However, strong limitations are present in the case of determinations at the ultra-trace levels: during the evaporation process, loss of analytes or external contamination can occur considering that ultra-clean conditions cannot be reached under a chemical hood (for safety reasons, a laminar flow hood cannot be employed when fluorine evaporation is performed). Boric acid (H_3_BO_3_) is also widely used to mask HF after MW-assisted digestion. However, the addition of H_3_BO_3_ may induce spectral interferences [18,19] and an overall increase of the background signal during ICP-MS measurements due to the increased matrix load of the digest and the likely sample contamination with impurities of the used chemicals [20]. Finally, magnesium (Mg) is proposed as an additive to inhibit the formation of insoluble Al fluorides [16,21]. Further methods are also reported to avoid atmospheric contamination or contamination related to impurities in added chemicals, based on the control of the sample dryness during evaporation [22] and of the pressure inside the vessel (e.g., open-vessels) [23,24] during MW digestion. Nevertheless, these procedures may lead to unreproducible results owing to the absence of agreement in the literature regarding the recommended experimental conditions (e.g., minimum sample mass to be digested).

Considering the drawbacks evidenced by existing methods, the development of a new fluoride removal protocol may be favorable if safer, cleaner and more controlled conditions are achieved, maintaining the same HF displacement efficiency.

Based on these considerations, herein, we propose a protocol to cleanly and efficiently remove HF from digestion solutions. The proposed procedure is executed using a sealed “vessel-inside-vessel” system [25] by performing two additional MW cycles after conventional HF-based digestion. As we will show, with this strategy, HF migrates from the inner PFA vessel (containing the solid sample to be digested and the acid mixture) to the PTFE outer solution (used as a sink for HF wastes). Water and concentrated boric acid (H_3_BO_3_) are tested as scavengers for the outer solution. A careful evaluation of the eventual cross-contamination effects is also presented. Finally, a certified soil reference material is digested to validate the proposed method.

## 2. Materials and Methods

### 2.1. Reagents

Ultrapure hydrofluoric acid (50% in water, Sigma-Aldrich, St. Louis, MS, USA) and ultrapure nitric acid produced by sub-boiling distillation [26] from commercial HNO_3_ (65% pure, Carlo Erba, Milan, Italy) were used for sample digestion. Hydrogen Peroxide (for trace analysis, ≥30%, Sigma-Aldrich) was used for soil moistening and organic matter decomposition. Ultrapure water was used for the preparation of each solution used and was produced with a Sartorius Arium mini plus UV Lab Water System. Saturated H_3_BO_3_ solutions were obtained by dissolving the solid (99.8%, Carlo Erba, Milan, Italy) in ultrapure water. Standard fluoride solutions (1, 5, 10 and 15 mg L^−1^) for IC analysis were obtained by dilution from a 1000 mg L^−1^ standard solution (Merck, Darmstadt, Germany). A multi-elemental standard solution (10 mg L^−1^ for Ag, Al, Ba, Be, Bi, Cd, Co, Cr, Cs, Cu, Ga, In, Li, Mg. Mn, Mo, Ni, Pb, Rb, Sr, Ti, V and Zn, 100 mg L^−1^ for Ca, K, Fe and Na; Sigma-Aldrich) was used for ICP-MS standard preparation and for samples preparation in cross-contamination tests. A certified soil from the National Institute of Standard & Technology (NIST 2710, Gaithersburg, MD, USA) was used as the Standard Reference Material (SRM) for protocol validation.

### 2.2. MW-Assisted Digestion and HF Evaporation Protocol

**Figure 1:**
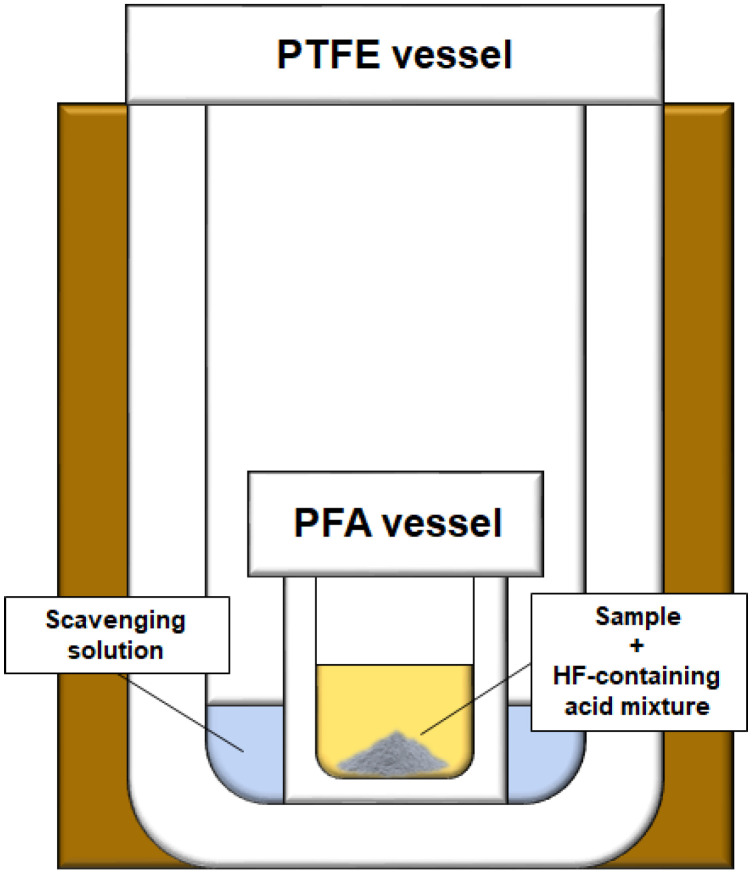
Schematic representation of the vessel-inside-vessel system used in the present work.

An ETHOS One (Milestone MLS, Bergamo, Italy) MW digestion system equipped with 6 PTFE vessels (internal volume ~80 mL) was used for all MW-assisted acid digestion reported in this work. A loosely tightened PFA vessel (Savillex, Minneapolis, MN, USA) with a round-shaped bottom interior (internal volume 5 mL) containing the digestion solution was placed inside each PTFE vessel. A scavenger solution was eventually added outside the PFA container (Figure 1). Three different conditions were evaluated: (i) the absence of a scavenging solution, (ii) 2 mL of ultrapure water and (iii) 2 mL of saturated H_3_BO_3_ solution as scavenging agents. Six samples were always loaded into the six-position carousel for all the reported experiments (one position is always dedicated to a blank solution) to guarantee the same conditions inside the reactor for all batches. The uniform power feeding distribution was ensured by keeping the carousel under constant rotation and filling all vessels with solutions possessing an analogous composition, thus having the same absorption behavior as microwaves. All quantitative results in this work (i.e., trace element and fluoride determination) are expressed as mean value ± two times the standard deviation determined over five replicated samples according to this batch configuration.

**Figure 2:**
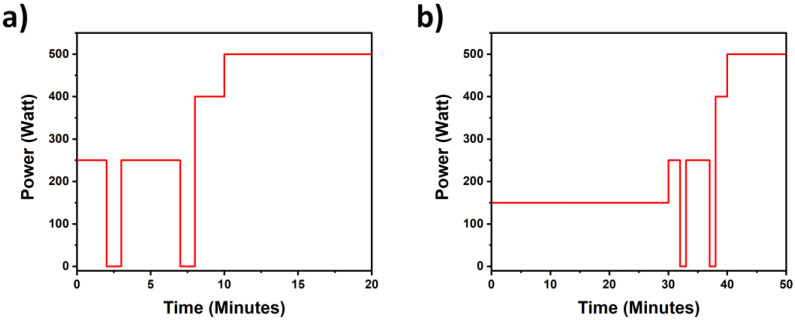
Microwave power programs used for (**a**) HF evaporation and (**b**) soil digestion.

The optimized sample digestion and HF displacement tests were conducted as follows:

- First cycle (sample digestion): 2 mL of HF were placed inside the PFA container, eventually together with the solid sample to be digested. The scavenging solution (2 mL) was placed in the external PTFE vessel. The application of both MW power programs shown in Figure 2a,b, was assessed for this step.
- Second cycle (first HF evaporation step): 1 mL of HNO_3_ was added into the PFA vessel to favor HF evaporation, and the scavenging solution was replaced with a fresh one. The MW power program depicted in Figure 2a was always used for this process.
- Third cycle (second HF evaporation step): an identical repetition of the second cycle which aimed to completely remove residual HF.

As mentioned above, two different MW power programs were developed in the present work. Figure 2a show the power program used for all HF evaporation cycles and the first preliminary digestion tests, while Figure 2b reports the MW program involved in the validated protocol for the dissolution of the certified soil NIST 2710 (for the first cycle only). The evaporation program involves a preliminary warm up at 250 W (6 min) and 400 W (2 min) before the 500 W treatment for 10 min. Intermediate steps at 0 W for 1 min were intended to favor the thermalization inside the PTFE and the PFA vessel. The dissolution program was instead composed of the first irradiation of 150 W for 30 min to properly dissolve the solid matrix [27], then the HF evaporation protocol was carried out as reported in Figure 2a.

The control of the MW process by temperature measurement was intentionally avoided as the temperature probe in vessel-inside-vessel systems cannot monitor the condition of the digestion solution but only that of the external scavenging solution. This constraint may lead to inaccurate or non-reproducible conditions if a temperature program is applied.

After each MW cycle, the solutions from both the inside and outside vessels were collected for fluoride determination by Ion Chromatography (IC) analysis using a Metrohm ECO IC equipped with a Metrohm 813 compact autosampler (Metrohm, Varese, Italy).

### 2.3. Certified Soil Digestion and ICP-MS Analysis

The NIST 2710 soil digestion procedure was adapted from the literature [27] to fit our vessel-inside-vessel strategy: 50 mg of certified soil were placed inside the PFA vessel and moistened with 1 mL of H_2_O_2_; the mixture was heated up on a hot plate at 50 °C for 30 min, under a laminar flow hood, to decompose most of the organic material. After the moistening procedure, 1 mL of HF and 1 mL of HNO_3_ were added to the PFA vessel, and the digestion program (Figure 2b) was applied. Then, hydrofluoric removal evaporation was performed as described above by performing two additional cycles (see MW program in Figure 2a).

After the mineralization/HF evaporation cycles, the sample solutions were transferred in low-density polyethylene (LDPE) bottles and diluted to 30 g with ultrapure water and then again, a dilution of 1:100 was performed before instrumental analysis. Prior to their use, the LDPE bottles were thoroughly cleaned and decontaminated by a 3-stage procedure involving prolonged washing with a detergent solution (4 mL L^−1^ Nalgene L900) and then with a 2% wt. HNO_3_ solution (see details in [25]).

A Thermo Scientific ICAP Q inductive coupled plasma mass spectrometer (ICP-MS, Thermo Fisher Scientific, Milan, Italy) was used to determine the concentration of 10 trace elements (Ag, As, Ba, Cd, Cu, Ni, Pb, V, Sb, Zn). Measurements were performed using a He-collision cell in kinetic energy discrimination (KED) mode.

### 2.4. Cross-Contamination Experiments

Cross-contamination tests were performed to evaluate the migration of trace elements from the sample solution to the scavenging one and vice versa. Both tests were performed by adding an aliquot of the multi-elemental standard solution used for ICP-MS analysis in the digestion solution and scavenger, alternatively. A total of 27 elements were analyzed (Ag, Al, Ba, Be, Bi, Ca, Cd, Co, Cr, Cs, Cu, Fe, Ga, In, K, Li, Mg. Mn, Mo, Na, Ni, Pb, Rb, Sr, Ti, V and Zn).

Tests were conducted as follows:

- Inside-to-Outside (i.e., sample-to-scavenger) migration: 100 µL of the multi-elemental standard solution were added to the hydrofluoric acid contained in the inner PFA vessel. Once the digestion/evaporation protocol was completed, the digestion solution was transferred to LDPE bottles and diluted to 20 g with ultrapure water to obtain a final concentration of 50 µg L^−1^ for the selected elements (500 µg L^−1^ for Ca, Fe, K and Na) in case of complete recovery. The obtained samples were then analyzed by ICP-MS.
- Outside-to-Inside (i.e., scavenger-to-sample) migration: 100 µL of the multi-elemental standard solution were added to ultrapure water in the outside vessel. Once the digestion/evaporation protocol was completed, the digestion solution was transferred to LDPE containers, diluted to 20 g with ultrapure water and analyzed by ICP-MS.

## 3. Results and Discussion

### 3.1. Proof of Concept and Scavenger Effect

The idea behind the proposed hydrofluoric acid removal method is to induce its evaporation directly inside a sealed PTFE vessel rather than using an open-vessel system (as is normally reported in the literature). To achieve this goal, a vessel-inside-vessel system was conceived (Figure 1): a small PFA vessel (5 mL), containing the HF-based digestion solution, is placed inside a PTFE vessel. During MW irradiation cycles, a relevant fraction of HF evaporates and tends to occupy all the available volume, which is represented by the small PFA head space (about 3 mL) and by the large volume inside the sealed PTFE vessel (about 70 mL): HF migration from the inner to the outer vessel is possible because the first one is only loosely tightened. Once the heating cycle is finished and the vessels are cooling down, the migrated HF condenses outside the PFA vessel and accumulates into the outer scavenging solution. The proposed strategy aims to (i) eliminate atmospheric contaminations due to the exposure of the digestion solution to air under a chemical hood (see open vessel evaporation), (ii) perform all manipulations under a laminar flow hood and (iii) eliminate contaminations related to impurities present in chemical additives used to mask or remove HF (here only ultrapure distilled HNO_3_ is added to the sample solution). Thus, the potential overall benefit of this proposed strategy is that it paves the way to perform determinations at the ultra-trace level even when the utilization of HF is mandatory.

Preliminary tests were conducted to achieve the proof of concept of the expected process and evaluate its effectiveness. Different experimental conditions were tested by varying the content of the outer PTFE vessel. Three different conditions were tested: (i) the absence of a scavenging solution, (ii) 2 mL of ultrapure water and (iii) 2 mL of saturated H_3_BO_3_ solution as scavenging agents. The latter is expected to act as an actual scavenger: the reaction with HF that leads to the formation of HBF_4_ species may possibly shift the evaporation equilibrium and accelerate the migration process. Each experiment was conducted as reported in Section 2.2. Three irradiation cycles were performed (following the power program reported in Figure 2a and solutions both from the inner and the outer vessels were collected after each cycle to determine their fluoride content.

**Figure 3:**
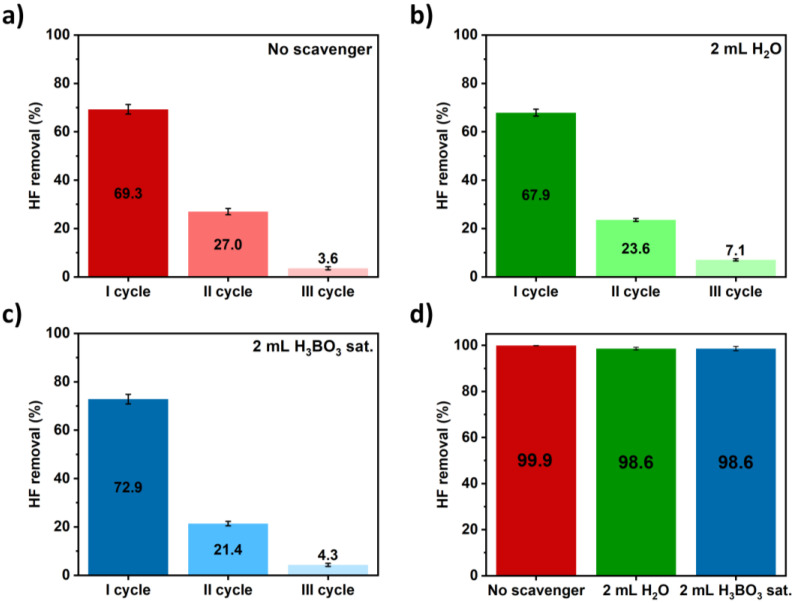
Comparison of HF removal efficiencies obtained using different experimental conditions outside the PFA vessel. (**a**–**c**) HF removal after each cycle using (**a**) no scavenger, (**b**) H_2_O and (**c**) H_3_BO_3_ as scavenging solutions. (**d**) A comparison of total HF removal and the entire MW protocol (three cycles).

The obtained results are summarized in Figure 3. As can be noticed in Figure 3a–c, most of the hydrofluoric acid (~70%) is lost during the first irradiation cycle regardless of the presence or the nature of the scavenging solution. This proves that HF migration occurs and is a robust and fast process (20 min MW program). However, further cycles are necessary to achieve the goal of a nearly 100% removal of HF. The complete elimination of HF was achieved after three cycles, again regardless of the experimental condition tested (Figure 3d): the highest removal was obtained in the absence of scavenging solutions (99.9 ± 0.1%), while no differences were found when using ultrapure water or an H_3_BO_3_ saturated solution. In both cases, slightly lower removal was observed (98.6 ± 0.6%).

All these findings suggest that the mechanism behind hydrofluoric acid migration is merely physical: the temperature reached inside the inner vessel allows HF evaporation which then tends to occupy all the accessible volume, i.e., it significantly migrates to the outer vessel. Such a mechanism fits very well with the observation that the variation of the chemistry outside the digestion vessel has no positive or negative effects on the migrated amounts. The slightly higher migration efficiency observed when using no scavenging solutions could be explained considering that microwaves can only be captured by the HF inner solution, which is then consistently overheated.

In preliminary digestion experiments carried out on quartz powders, it was noticed that, in the absence of any outer scavenging solution, incomplete dissolutions were observed. This evidence could be ascribed to too rapid HF evaporation in the first cycle. For this reason, all further experiments were always conducted using 2 mL of ultrapure water in the outer vessel: the use of boric acid was discarded because it does not improve the removal efficiency, and, therefore, it is possible to avoid the use of unnecessary chemicals that could induce unwanted contaminations. Such choice also allowed for the handling of diluted HF solutions as wastes after digestion, which can be more safely dismissed or recycled for other analytical or non-analytical purposes (e.g., surface cleaning, etching and nanostructuring of semiconductors, biological applications [28,29,30,31,32]). This process, eliminates dangerous wastes and thus perfectly fits with one of the principles of green analytical chemistry [33].

### 3.2. Trace Elements Migration Tests: Evaluation of Cross Contamination Processes

In order to rule out the migration of trace elements, in addition to that of HF, cross-contamination tests were performed to evaluate the transport of analytes (i) from the digestion solution to the scavenger and (ii) from the scavenger to the digestion solution at the end of entire digestion/evaporation protocol (see details in Section 2.4). The investigation of the first process aimed to evaluate the possible loss of analytes during the digestion/evaporation protocol, whereas the study of the second one aimed to assess the potential contamination from the scavenging solution. Although ultrapure water was used as an external sink for HF (and so this issue should not be relevant), it is well known that the porous nature of the PTFE vessel causes relevant memory effects since gases and other contaminants are prone to be trapped and released during digestion heating cycles [12,34].

**Figure 4:**
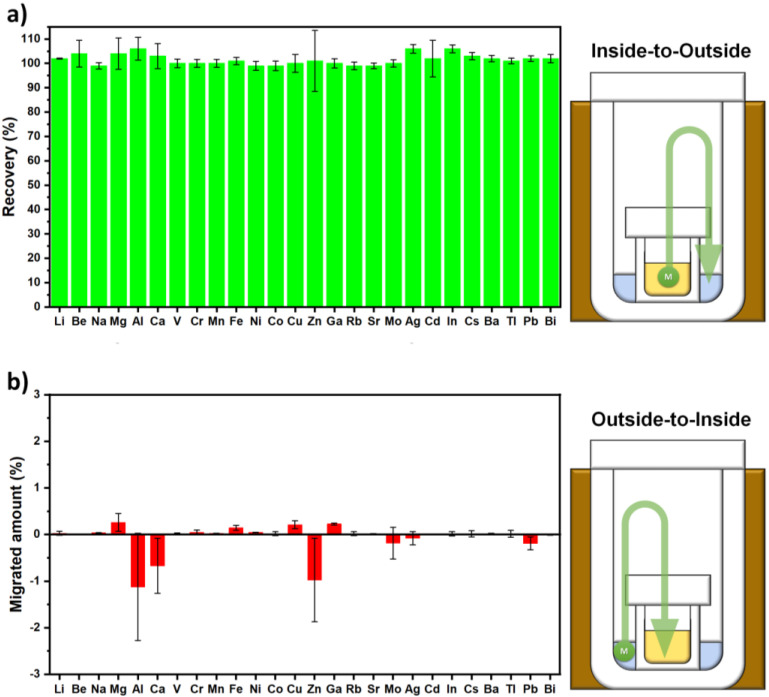
Recoveries obtained for 27 selected trace elements when analyzing the digestion solution after cross-contamination experiments: (**a**) Inside-to-Outside migration test and (**b**) Outside-to-Inside migration test.

As reported in Figure 4a, complete elemental recovery (not statistically different from 100%) was achieved for all the 27 elements investigated in this work when spiking the digestion solution. This means that no loss of analytes occurred during the entire MW treatment (three cycles). Concerning the reverse contamination experiment (outside to inside), no noticeable elemental contamination was observed when the scavenging solution was spiked with the elemental multistandard; all the elements showed a migration rate not significantly different from the concentration observed for the pure acids being used (Figure 4b). All these data prove that the proposed method is not only suitable for HF removal but it is also free from contamination during digestion and evaporation.

### 3.3. Certified Soil Digestion and Validation

Once we had demonstrated that the proposed approach allows for the easy and fast removal of HF from digestion solution, we validated the analytical protocol by digesting and analyzing a certified siliceous soil (NIST 2710).

**Figure 5:**
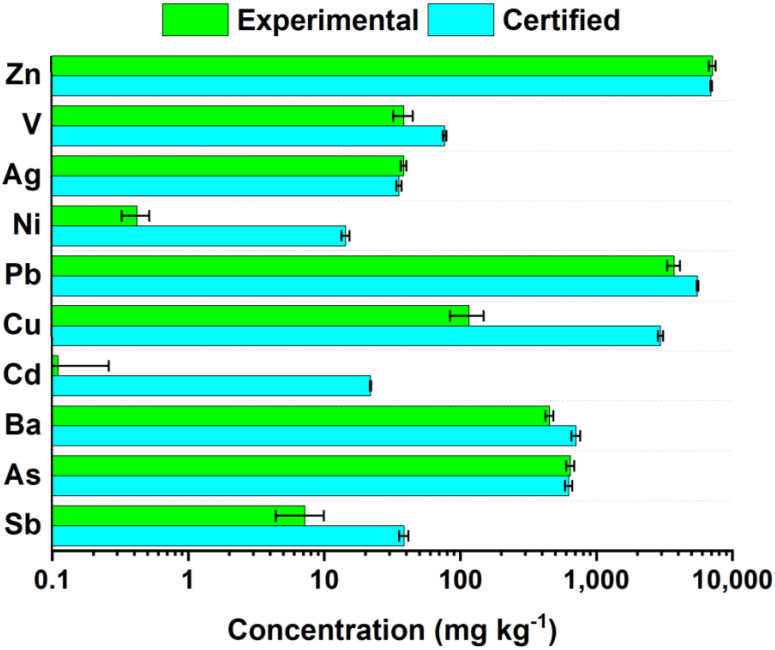
Comparison of certified (light blue bars) and experimental (green bars) trace elements concentrations found in NIST 2710 by applying three times the MW power program reported in Figure 2a.

The MW program depicted in Figure 2a was applied for three cycles, as for experiments reported in previous paragraphs. The obtained solution was diluted and then analyzed by ICP-MS (Figure 5). These data clearly show that some problems were encountered for several elements: very poor recoveries were observed for Sb, Cd, Cu and Ni, whereas only As, Ag and Zn were completely recovered (recoveries = 103, 108 and 102%, respectively). As a matter of fact, it was observed in all vessels that with this protocol, an incomplete dissolution of both siliceous and organic fractions was always obtained. This partial dissolution (which, in turn, is the cause of poor recoveries) was ascribed to two main issues: (i) the substantial HF loss (about 70%) during the first heating cycles was probably too fast to allow the complete dissolution of silicates; (ii) HF and HNO_3_ are probably not suitable for the complete digestion of the organic fraction.

To overcome such problems, the soil dissolution procedure was revised. First of all, according to the protocol used by Matusiewicz et al. for the digestion of the NIST 2710 SRM [27], pre-digestion moistening with 30% H_2_O_2_ was conducted at around 50 °C on a hot plate under a laminar flow hood to decompose organic matter. Secondly, 2 mL of a 1:1 mixture of HF and HNO_3_ was added to the moistened soil, and the MW-assisted digestion procedure was carried out by revising the MW power program. A low power step (150 W for 30 min) was introduced to retard HF evaporation (see Figure 2b).

**Figure 6:**
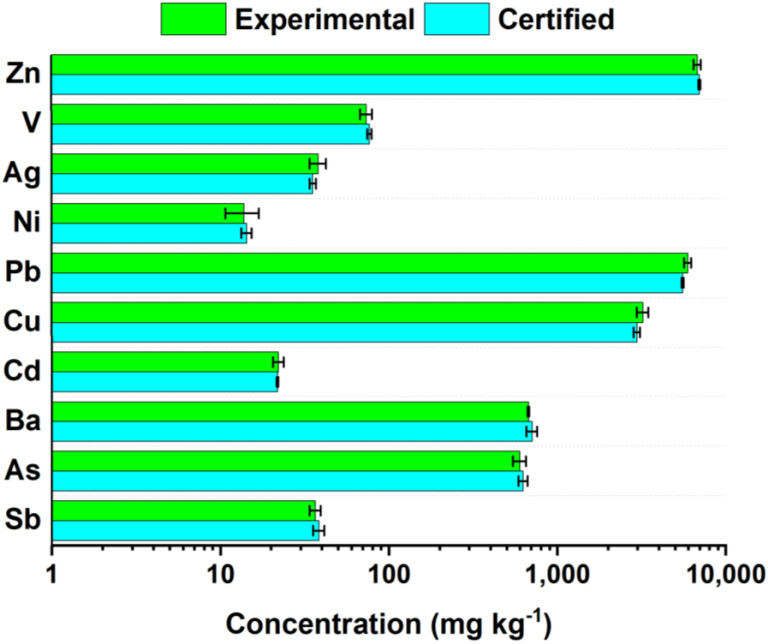
Comparison of certified (light blue bars) and experimental (green bars) trace elements concentrations found in NIST 2710 by applying the revised analytical protocol (pre-digestion moistening with H_2_O_2_ followed by the revised MW-assisted digestion, see Figure 2b).

After performing this modified digestion cycle and two subsequent HF evaporation steps, the solid sample was completely dissolved, and a complete recovery was achieved for all certified trace elements. As depicted in Figure 6, recoveries in the range of 95–108% (average recovery = 100 ± 6%) were obtained. Moreover, the first digestion step was revised to slow down HF evaporation; a 98.4% HF migration efficiency was still observed after the three MW cycles.

These results demonstrate that the proposed MW-assisted digestion/HF evaporation protocol was validated for the analysis of siliceous materials as well as for the complete removal of HF from the digestion solution.

## 4. Conclusions

In the present work, a vessel-inside-vessel method consisting of a fixed high-power irradiation treatment (500 W) for HF evaporation was proposed and proved effective for the fast, safe and especially clean removal of hydrofluoric acid. This last issue represents the major advantage of the proposed protocol, as HF removal is performed in sealed vessels, thus ruling out any possible contamination in this crucial step. This protocol is therefore compliant with elemental determination at ultra-trace levels when the utilization of HF is mandatory.

It was determined that the mechanism behind hydrofluoric acid removal is purely physical: HF evaporation in the digestion vessel migrates into the large volume of the outer PTFE vessel and, after cooling down, condenses into the scavenging solution. The presence of ultrapure water as a scavenging solution in the PTFE vessel allows for better control of the evaporation process and the handling of diluted hydrofluoric acid solutions, which can be safely dismissed or collected and recycled for other applications at the end of each MW cycle. This side feature is perfectly in line with the principles of green analytical chemistry, making the proposed method even more attractive.

Finally, the effectiveness of the MW-assisted digestion/evaporation protocol was validated on the NIST 2710 certified soil.
